# Temporal Kinematic Differences between Forward and Backward Jump-Landing

**DOI:** 10.3390/ijerph17186669

**Published:** 2020-09-13

**Authors:** Datao Xu, Xuanzhen Cen, Meizi Wang, Ming Rong, Bíró István, Julien S. Baker, Yaodong Gu

**Affiliations:** 1 Faculty of Sports Science, Ningbo University, Ningbo 315211, China; xudatao3@gmail.com (D.X.); cenxuanzhen@outlook.com (X.C.); 2 Savaria Institute of Technology, Eötvös Loránd University, 9700 Szombathely, Hungary; nbuwangmeizi@aliyun.com; 3 Faculty of Engineering, University of Szeged, 6724 Szeged, Hungary; biro-i@mk.u-szeged.hu; 4 Centre for Health and Exercise Science Research, Hong Kong Baptist University, Hong Kong 999077, China; jsbaker@hkbu.edu.hk

**Keywords:** backward jump-landing, non-contact ACL injuries, knee flexion, temporal kinematic, statistical parametric mapping (SPM)

## Abstract

Backward jump-landing during sports performance will result in dynamic postural instability with a greater risk of injury, and most research studies have focused on forward landing. Differences in kinematic temporal characteristics between single-leg and double-leg backward jump-landing are seldom researched and understood. The purpose of this study was to compare and analyze lower extremity kinematic differences throughout the landing phases of forward and backward jumping using single-leg and double-leg landings (FS and BS, FD and BD). Kinematic data were collected during the landing phases of FS and BS, FD and BD in 45 participants. Through statistical parametric mapping (SPM) analysis, we found that the BS showed smaller hip and knee flexion and greater vertical ground reactive force (VGRF) than the FS during 0–37.42% (*p* = 0.031), 16.07–32.11% (*p* = 0.045), and 23.03–17.32% (*p* = 0.041) landing phases. The BD showed smaller hip and knee flexion than the FD during 0–20.66% (*p* = 0.047) and 0–100% (*p* < 0.001) landing phases. Most differences appeared within a time frame during the landing phase at 30–50 ms in which non-contact anterior cruciate ligament (ACL) injuries are thought to occur and are consistent with the identification of risk in biomechanical analysis. A landing strategy that consciously increases the knee and hip flexion angles during backward landing should be considered for people as a measure to avoid injury during the performance of this type of physical activity.

## 1. Introduction

Anterior cruciate ligament (ACL) injuries occur commonly in sports, such as basketball, volleyball, football, etc., 70% of which are non-contact injuries [1,2,3]. These injuries often affect athletic performance in the competitive arena and have negative effects on careers [4]. Previous studies have observed that non-contact ACL injuries mainly occur during the performance of sport actions such as landing, takeoff, and lateral cuts [5,6,7,8]. Landing from height is one of the most important risk factors for ACL injury, especially during jump-landing activities. Different landing patterns may have great impact on the loadings associated with the ligaments and joints of the lower limbs and an unreasonable landing mode usually causes functional valgus collapse [9,10]. Functional valgus collapse is a movement composed of hip and knee motion in the sagittal and frontal planes and is believed to increase the risk of non-contact ACL injury [11,12,13]. A successful landing task requires enough muscle strength in combination with joint and posture stability to protect the lower limbs from injury and an unstable landing is likely to cause lower limb damage [14]. At the same time, the knee and hip joint demonstrate a smaller flexion angle when adopting a more rigid landing position [9]. A smaller flexion could cause the lower limbs to endure a greater ground reaction force load rate, knee extension torque, knee abduction torque, and forward sheer force of the tibia, which are significant risk factors for ACL injury [15].

Jump-landing is one of the most basic techniques used in sport and has been confirmed as a mechanism for non-contact ACL injury [16,17,18]. The jump-landing movement mainly consists of landing from a variety of directions on both legs or on a single leg. Postural stability is a significant risk factor for damage during landing. Dynamic postural stability has been defined as an ability to keep dynamic balance by maintaining a projected center of mass within the limits of the point of support [19]. Dynamic postural stability depends on the comprehensive feedback and movement strategy of the hip, knee, and ankle, and poor dynamic postural stability will lead to greater energy impact on the lower limb motor chain. The knee joint is a joint capsule with multiple joints, which is easily damaged during the energy impact and transmission of the lower limb motor chain [20,21]. During jump-landing movements, the measurement of dynamic postural stability is critical to determining predictors of performance, assessing lower extremity musculoskeletal injuries, and injury prevention through the study of injury risk factors [22,23,24]. Previous studies have shown that dynamic postural stability can be a risk factor for knee injuries [25,26]. Therefore, dynamic postural stability is also considered as an important factor in the risk of ACL injury. Previous studies show differences in dynamic postural stability for the forward direction compared with that for all other landing directions, and time to stabilization showed significantly longer times for landings from the forward and backward directions [19,27]. Therefore, there will be a greater risk of injury when making a backward jump-landing during sports performance, because the decline of proprioception and vision limitation will cause greater dynamic postural instability.

Consideration needs to be given to the timing of jumping techniques, which may affect the chance of damage during comparisons between forward or backward, single- and double-leg landing tasks [28]. Previous studies have already indicated that ACL injuries are most likely to happen during the initial 30–50 milliseconds (ms) of foot contact with the ground when performing a landing task [12]. However, current kinematic analyses tend to concentrate on the peak joint angle and the range of motion, which coincides with the time series when the risk of injury is highest. Although this method is extensively accepted, it still should be considered that the approach needs to be improved. This method of comparing the range of motion and the peak of the joint angle ignores the significance of time synchronization that may occur during the entire landing phase. Statistical parameter mapping (SPM) is a method for time series statistical analysis of continuous data over a period of time. It can test and analyze the statistical differences of data changing over time during the entire performance [29]. Through the statistical analysis of the over-simplified vector trajectory, an objective and comprehensive statistical conclusion is obtained, which can objectively guide the analysis of complex biomechanical systems [30]. Considering the one-dimensional characteristic of the joint angle changing with time, statistical parameter mapping (SPM) 1D was used for statistical analysis of the data.

Therefore, based on the few studies that have focused on backward landing biomechanics, the purpose of this study was to compare and analyze sagittal- and frontal-plane ankle, knee, and hip motions and vertical ground reaction force (VGRF) throughout the landing phases of forward and backward jumping using a single-leg and double-leg landing (FS and BS, FD and BD) using statistical parametric mapping (SPM) 1D analysis and conventional discrete kinematic analysis. We hypothesized that compared to forward landing, backward landing will exhibit smaller flexion and greater abduction angles and VGRF due to greater dynamic posture instability. To maintain balance, the knee and hip will also show a smaller joint range of motion (ROM) and peak joint angle during the backward landing phase. The ankle joint will also exhibit greater dorsiflexion and adduction angles to maintain balance, and these differences are distributed within a time frame of 30–50 ms after initial ground contact.

## 2. Materials and Methods

### 2.1. Participants 

A total of 45 subjects (age: 23.1 ± 1.7 years, height: 180.0 ± 4.9 cm, weight: 77.2 ± 7.7 kg, BMI: 23.8 ± 1.8 kg/m^2^) participated in this experiment. All subjects were amateur athletes and performed a variety of physical exercise for at least 30 min at least 3 times a week (basketball, volleyball, football). None of the subjects had a history of lower extremity surgery. No lower extremity injuries were reported in the six months before the trial and no medical problems that might affect their performance were reported. Prior to the experiment, all subjects were informed of the purpose, requirements, procedures, and conditions of the study, and all provided written informed consent. The study was approved by the Ethics Committee of the University.

### 2.2. Instrumentation

**Figure 1:**
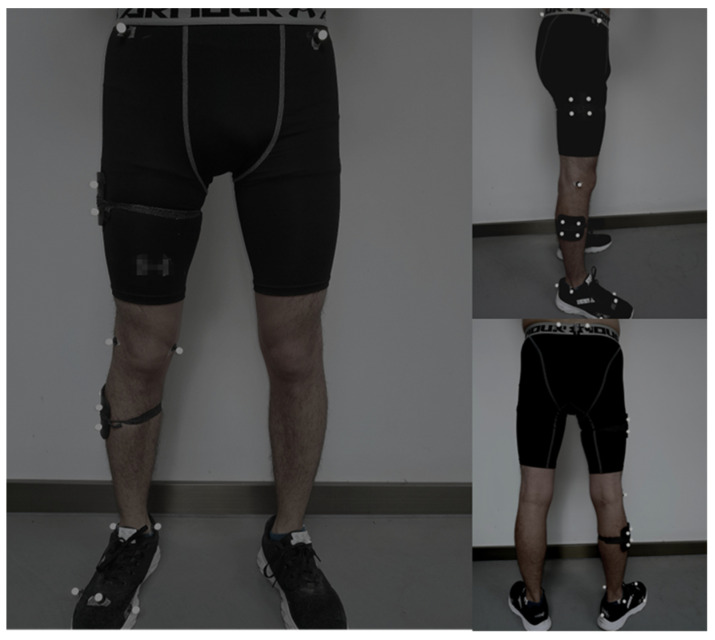
Illustration of the front, side, and back marker placements.

Before the motion capture data collection, participants were asked to wear the same shoes to eliminate the potential error caused by different shoes during landing. A Vicon motion capture system (Vicon Metrics Ltd., Oxford, UK) with eight cameras was used to capture the motion during the landing phase, and the sampling frequency was set at 200 Hz. According to the experimental requirements and previous data acquisition, 20 reflective markers (diameter: 12.5 mm) were attached to the participants’ lower limbs to track movement. Figure 1 shows the marker placement. The marker locations included: right and left anterior superior iliac spine, left and right posterior superior iliac spine, medial and lateral condyle, medial and lateral malleolus, first and fifth metatarsal heads, distal interphalangeal joint of the second toe. Tracking clusters were placed on the middle and lateral thigh, shank, and right heel [31].

The static experiment collected data on the participants in the anatomical neutral position and used this dataset as initial coordinates. The X-axis plane is defined as the sagittal plane (flexion and extension), the Y-axis plane as the frontal plane (adduction and abduction), and the Z-axis plane as the horizontal plane (internal rotation and external rotation). Kinetic data were obtained through an in-ground force plate (AMTI, Watertown, MA, USA) and data were sampled at a frequency of 1000 Hz. Vicon Nexus 1.8.6 software was used to synchronously collect kinematics and ground reaction data.

### 2.3. Procedure of Forward and Backward Jumping with Single-Leg and Double-Leg Landings

Before the formal experiment, participants were asked to wear uniform tights and shoes, warm-up for 10 min at a speed of 8 km/h on a treadmill, and then perform full muscle stretching. At the end of the warm-up, the participants practiced the whole test movement until they became familiarized with testing procedures. Subjects were allowed to conduct three practical experiments. Participants started from a stationary standing position at a height of 40 cm from the ground, and the starting point was 70 cm from the center of the platform [27]. During forward single-leg landing (FS), participants were asked to jump to the center of the force plate with both feet, so that the dominant leg (the preferred leg in a daily exercise that is better suited for landing or kicking a ball) would land on the force plate for support and maintenance of balance. Participants were asked to maintain their balance for five seconds, starting at the point of contact with the ground. During forward double-leg landing (FD), participants were asked to land with the dominant leg inside the force plate and the other leg outside. The dominant leg data were then collected. During backward single-leg and double-leg landing (BS and BD), participants turned their back to the force plate and made a jump-landing to the rear, with the remaining requirements consistent with those outlined for forward landing.

During landing performance, the center of gravity becomes unstable, causing the body to oscillate from side to side. If the participant tried to maintain balance by holding on to the ground or other objects with his hands the experiment was considered as a failure. Data were collected on five successful landings, and a total of 20 sets of data were obtained for the four jumping performances. There was a 30 s rest period observed between each landing to avoid fatigue of participants caused by continuous jump-landing, which would affect the accuracy of data capture.

### 2.4. Data Collection and Processing

The initial contact point is defined as the vertical ground reaction force (VGRF) exceeding 10 N [32]. Data collection begins two seconds before the initial contact with the ground and ends three seconds after the contact with the ground, totaling 5 s of data collection. According to Winter’s description of the selected frequency of the filter [33], the residual analysis of VGRF was carried out in the subsets to determine the most appropriate signal-to-noise ratio. Finally, the data of kinematics and VGRF are filtered by 10 and 20 Hz fourth-order zero-phase lag Butterworth low-pass filters. Then data were exported into MATLAB R2019a (The MathWorks, MA, United States), and the written script was used to process the data. The landing phase is defined as the point from initial ground contact to maximum knee flexion. 

For SPM analysis, the generation of a separate integration curve for each task before performing SPM analysis was performed. All kinematic and VGRF data of the landing phase were extracted, and the data points were expanded into a time series curve of 101 data points (representing 0–100% of the landing phase) with a custom MATLAB script. For the traditional discrete variable analysis, a MATLAB script was written to extract the peak VGRF and peak angle points of the knee, hip, and ankle joint in the sagittal plane and frontal plane during the landing stage. Once completed, the corresponding ROM of joint angles was calculated. The vertical instantaneous loading rate (VILR) was calculated as peak VGRF divided by the time it takes to reach VGRF [34].

### 2.5. Statistical Analysis

Prior to statistical analysis, the Shapiro–Wilk normality test was performed on all data. The Wilcoxon matched-pairs signed-rank test was conducted for non-parametric data if nonconformity was observed. For traditional discrete variable analysis, a single repeated-measures ANOVA was employed to test differences of peak VGRF and VILR, peak joint angles, and ROM of joint angles between the knee, hip, and ankle. All traditional discrete variable analyses were performed using SPSS 24.0 for Windows^TM^ software (SPSSs Inc, Chicago, IL, USA), and 0.05 (α = 0.05) was set as being statistically significant. Cohen’s d effect sizes (ES) were also calculated and divided into three classes of benchmark (small: ES > 0.2; medium: 0.2 < ES < 0.5; large: ES > 0.8) [35]. For SPM analysis, the joint kinematics and VGRF time series curve were marked as 100%, we also carried out a paired-samples T-test in MATLAB to analyze FS and FD as well as BS and BD. The open-source SPM 1D script was used for the statistical analysis, and the significance threshold was 0.05 [29].

## 3. Results

**Figure 2:**
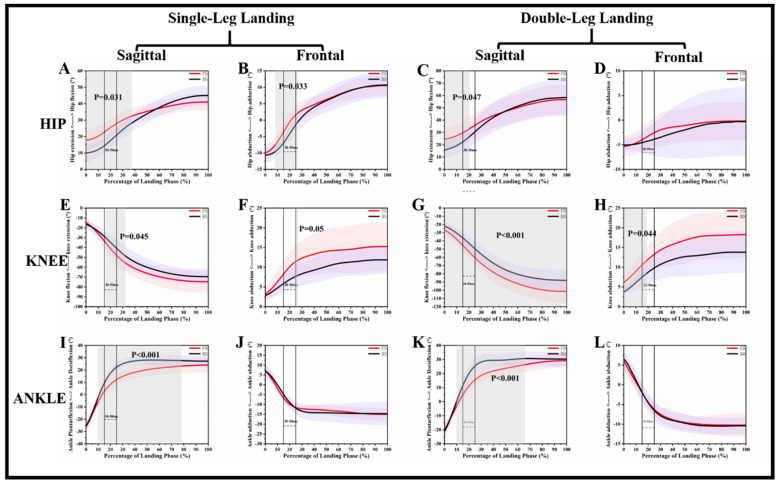
Descriptive results between forward landing and backward landing task statistical parametric mapping results, depicting the hip, knee, and ankle joint in the sagittal and frontal planes and single-leg landing and double-leg landing mean angle trajectories and standard error. The range of the two lines is the time frame between 30 and 50 ms time after landing. Gray shaded areas are statistically different (*p* < 0.05) between forward landing and backward landing during the landing phase. (**A**): Hip angle change in Sagittal plane during single leg landing; (**B**): Hip angle change in Frontal plane during single leg landing; (**C**): Hip angle change in Sagittal plane during double leg landing; (**D**): Hip angle change in Frontal plane during double leg landing; (**E**): Knee angle change in Sagittal plane during single leg landing; (**F**): Knee angle change in Frontal plane during single leg landing; (**G**): Knee angle change in Sagittal plane during double leg landing; (**H**): Knee angle change in Frontal plane during double leg landing; (**I**): Ankle angle change in Sagittal plane during single leg landing; (**J**): Ankle angle change in Frontal plane during single leg landing; (**K**): Ankle angle change in Sagittal plane during double leg landing; (**L**): Ankle angle change in Frontal plane during double leg landing. FS: forward single-leg landing; FD: forward double-leg landing; BS: backward single-leg landing; BD: backward double-leg landing.

**Figure 3:**
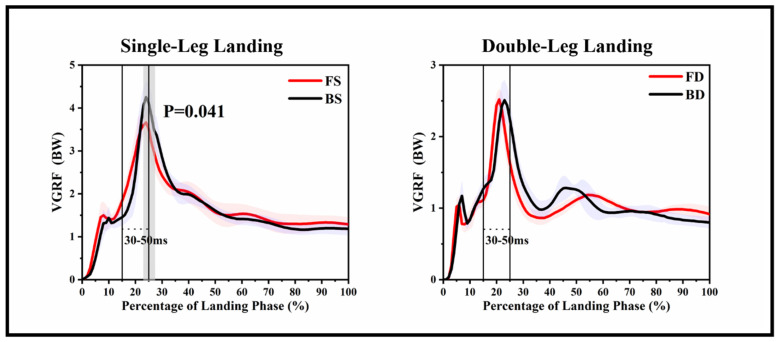
Descriptive results between forward landing and backward landing task statistical parametric mapping results, depicting the VGRF of single-leg landing and double-leg landing mean forces trajectories and standard error. The range of the two lines is the time frame between 30 and 50 ms time after landing. Gray shaded areas are statistically different (*p* < 0.05) between forward landing and backward landing during the landing phase. FS: forward single-leg landing; FD: forward double-leg landing; BS: backward single-leg landing; BD: backward double-leg landing.

Figure 2 and Figure 3 show the VGRF and kinematic differences of each landing phase in corresponding time series, and Table 1 and Table 2 show the results of the traditional discrete statistical analysis. 

For the hip joint, Figure 2A reveals that FS depicted a greater flexion angle than BS during the 0–37.42% landing phase (*p* = 0.031). Figure 2C reveals that FD depicted a greater flexion angle than BD during the 0–20.66% landing phase (*p* = 0.047). Figure 2B reveals that FS depicted a greater adduction angle than BS during the 8.33–26.37% landing phase (*p* = 0.033). Table 1 shows that FS and FD depicted a greater min-flexion angle than BS (*p* < 0.001) and BD (*p* < 0.001).

For the knee joint, Figure 2E reveals that FS depicted a greater flexion angle than BS during the 16.07–32.11% landing phase (*p* = 0.045). Figure 2G reveals that FD depicted a greater flexion angle than BD during the 0–100% landing phase (*p* < 0.001). Figure 2F reveals that FS depicted a greater adduction angle than BS during the 23.91–26.74% landing phase (*p* = 0.05). Figure 2H reveals that FD depicted a greater adduction angle than BD during the 0–18.72% landing phase (*p* = 0.044). Table 1 shows that FS and FD depicted a greater ROM-flexion angle than BS (*p* = 0.005) and BD (*p* = 0.008). 

For the ankle joint, Figure 2I reveals that FS depicted a smaller dorsiflexion angle than BS during the 9.92–78.01% landing phase (*p* < 0.001). Figure 2K reveals that FD depicted a smaller dorsiflexion angle than BD during the 9.90–65.82% landing phase (*p* < 0.001). There were no differences between forward and backward jumping landing on the frontal plane of the ankle. Table 1 shows that FS depicted a smaller max-flexion angle than BS (*p* = 0.019).

For the VGRF, Figure 3 reveals that BS depicted a greater VGRF than FS during the 23.03–17.32% landing phase (*p* = 0.041). There were no differences between FD and BD for VGRF. Table 2 shows that BS depicted a greater peak VGRF (*p* = 0.001) and VILR (*p* = 0.041) than FS.

## 4. Discussion

We hypothesized that compared to forward landing, performing backward landing will exhibit smaller flexion and abduction angles due to the greater dynamic posture instability. To maintain balance, the knee and hip will also show a smaller joint ROM and peak joint angle (peak flexion angle, peak extension angle, peak abduction angle, peak adduction angle) during the backward landing phase. At the same time, backward landing will show greater VGRF than forward landing. The ankle joint will also exhibit greater dorsiflexion and adduction angles to maintain balance, and these significant differences are basically distributed within a time frame of 30–50 ms after the initial ground contact. The results and findings of this study support this view in part. Consistent with our hypothesis, the SPM analysis demonstrated statistical differences between the hip and knee joints during the first part of the landing phase. However, SPM analysis showed no difference in the angle of the frontal plane of the ankle. For VGRF and VILR, the differences only were observed in single-leg landing.

Previous studies have shown that smaller flexion angles of the knee and hip during landing tasks will increase shear force, abduction, and the internal rotation moment of the knee, thereby increasing the risk of ACL injuries [9,36]. Our results show that BS and BD depicted a smaller knee and hip flexion angle than those of FS and FD, and these differences were significant, corresponding to the 16.07–32.11%, 0–100%, 0–37.42%, 0–20.66% landing period. Previous studies have also shown that a smaller knee flexion will cause greater VGRF, and the greater VGRF and VILR during landing were considered to increase the risk of ACL injury [37,38,39]. Consistent with our results BS depicted a greater VGRF than FS during the 23.03–17.32% landing phase (*p* = 0.041), and BS depicted a greater peak VGRF (*p* = 0.001) and VILR (*p* = 0.041) than FS. All differences appeared within a time frame of the landing phase at 30–50 ms, this is consistent with the time point of a high probability of ACL injuries [12]. Arms et al. found that as the flexion angle of the knee increases from 0° to 45°, the quadriceps force significantly increases the load on the ACL, but when the angle exceeded 60°, the quadriceps femoris muscle contraction had no significant effect on the ACL [40]. There is no doubt that our results are consistent with this, especially on the single-leg landing task, BS depicted a smaller ROM than that of FS (*p* = 0.005). Female athletes have a higher incidence rate of non-contact ACL injuries than that in male athletes, while female athletes have a smaller knee joint flexion angle in running, jumping, and other activities. It is possible that greater knee joint ROM during impact may allow a longer time to dissipate and control the ground reaction. Therefore, backward landing is likely to increase the risk of ACL injuries.

Backward landing shows more dynamic instability compared to landing in the other direction [19]. In the sagittal plane, BS and BD depicted a greater ankle flexion angle than FS and FD, and their differences were significant corresponding to the 9.92–78.01% (*p* < 0.001) and 9.90–65.82% (*p* < 0.001) landing period. This is very similar to the kinematics during landing in patients with chronic ankle instability (CAI). For CAI patients, the dynamic stability and knee flexion angle decreases, and ankle dorsiflexion angle will increase during the landing mission [41,42]. This is a similar landing mechanism even though there is no difference in ankle frontal plane kinematics for backward landing. For Figure 2I,J, we can clearly observe that the slope of the angle rising for backward landing in the first 30–50 ms after the initial contact is higher than that in forward landing. Our results also show that most of the significant differences in the kinematics of the knee and hip joints in the sagittal and frontal planes occurred at 30–50 ms of initial contact, for example, BS depicted a greater hip abduction angle than FS during 8.33–26.37% landing phase (*p* = 0.033). Previous studies have shown that ankle movements during landing affect the knee and hip joints [43]. The inadequate impact absorption of the ankle will increase the energy dissipation requirement of the proximal joint of the lower extremity, which will lead to the injury of the knee joint and the non-contact ACL injury [44,45,46].

Our results found that there are a lot of kinematic differences between forward landing and backward landing in the time series during the landing phase, but the results of traditional discrete analysis did not reveal this point. Especially, for the ankle joint, the traditional discrete analysis showed that there was no difference, and this finding needs to be taken seriously and researched further. Considering the significant difference in the sagittal plane of the ankle during the 30–50 ms period of initial contact, the impact on the knee and ankle joints after landing is an important factor to increase the risk of non-contact ACL injury. Therefore, the evidence of our research suggests that, to reduce non-contact ACL injury during backward landing, a landing strategy by increasing the flexion angle of the knee and hip should be proposed.

Undeniably, our study has some limitations. The participants in our experiment were only young males (healthy young males aged 19 to 26 years). Subsequent studies should consider other subjects, especially females, and previous studies have shown that female athletes differ significantly from men when landing. To accurately compare the time series curves between movements, it is necessary to normalize the landing phase length to 100%. The time required to complete the task is variable, and we acknowledge that recording the time series curve to 100% may mask some variability among subjects over time. At the same time, the frame rate used of our study was mostly focused on just 30–50 ms of data, therefore, it may have overlooked the effect of other periods of time throughout the landing on ACL injury. Therefore, even though the ACL injury is most likely to happen during the initial 30–50 ms, the differences in other phases of landing should also be taken into account in future studies.

## 5. Conclusions

In summary, this study compared and analyzed sagittal- and frontal-plane ankle, knee, and hip motions throughout the landing phases of forward and backward jumping using single-leg and double-leg landing. Within the time range of ACL injury, backward landing shows smaller knee and hip flexion angles, and forward landing shows smaller hip abduction and ankle dorsiflexion angles. BS show greater VGRF and VILR than FS does. Therefore, backward landing may increase the risk of non-contact ACL injury compared to that during forward landing. A landing strategy that consciously increases the knee and hip flexion angles during backward landing should be considered for people as a measure to avoid injury during the performance of this type of activity.

## Figures and Tables

**Table 1:** The hip peak joint angle and range of motion (ROM) of the joint angle during the landing phase between forward landing and backward landing.

				Forward Landing	Backward Landing	*p*
Hip	SP	MAX	SL	41.1 ± 5.2 *	45.0 ± 6.0 *	0.042
			DL	56.7 ± 12.3	58.3 ± 14.9	0.686
		MIN	SL	17.7 ± 6.4 *	9.9 ± 5.5 *	0.001
			DL	24.4 ± 9.1 *	15.8 ± 6.6 *	0.001
		ROM	SL	23.4 ± 4.8 *	35.1 ± 6.5 *	0.001
			DL	32.3 ± 7.3 *	42.5 ± 11.2 *	0.001
	FP	MAX	SL	10.5 ± 3.0	10.8 ± 3.9	0.839
			DL	0.2 ± 3.8	0.5 ± 6.3	0.740
		MIN	SL	−10.0 ± 2.3	−10.7 ± 2.3	0.406
			DL	−5.5 ± 2.1	−6.1 ± 2.0	0.173
		ROM	SL	20.5 ± 2.9	21.2 ± 5.11	0.527
			DL	5.6 ± 4.0	6.6 ± 5.4	0.583
Knee	SP	MAX	SL	−14.7 ± 4.7	−16.5 ± 4.4	0.192
			DL	−26.7 ± 5.8 *	−21.9 ± 5.3 *	0.006
		MIN	SL	−74.8 ± 11.2	−69.6 ± 7.7	0.074
			DL	−101.4 ± 13.8 *	−88.1 ± 12.8 *	0.001
		ROM	SL	60.0 ± 9.00 *	53.1 ± 7.1 *	0.005
			DL	74.7 ± 9.4 *	66.2 ± 11.3 *	0.008
	FP	MAX	SL	15.4 ± 6.0	12.0 ± 3.5	0.109
			DL	18.4 ± 5.8	14.0 ± 5.3	0.063
		MIN	SL	3.2 ± 2.1	2.8 ± 0.9	0.557
			DL	6.1 ± 2.4 *	3.7 ± 0.9 *	0.006
		ROM	SL	12.2 ± 4.3	9.2 ± 3.0	0.06
			DL	12.4 ± 3.8	10.3 ± 4.8	0.252
Ankle	SP	MAX	SL	24.3 ± 5.9 *	28.4 ± 4.5 *	0.019
			DL	29.1 ± 5.1	31.3 ± 4.8	0.161
		MIN	SL	−26.1 ± 3.5	−25.7 ± 4.4	0.747
			DL	−20.1 ± 3.7	−21.4 ± 4.7	0.337
		ROM	SL	50.9 ± 4.2	54.1 ± 7.0	0.053
			DL	49.1 ± 6.8	52.7 ± 7.4	0.123
	FP	MAX	SL	7.1 ± 3.3	7.1 ± 2.5	0.968
			DL	6.0 ± 2.4	6.6 ± 2.2	0.593
		MIN	SL	−15.3 ± 3.2	−15.4 ± 5.1	0.967
			DL	−10.8 ± 2.5	−10.7 ± 2.1	0.915
		ROM	SL	22.4 ± 4.5	22.4 ± 5.0	0.990
			DL	16.9 ± 1.6	17.3 ± 2.2	0.613

Note: SP: sagittal plane, FP: frontal plane, MAX: maximal, MIN: minimum, ROM: range of motion of joint angle, SL: single leg, DL: double leg. * Refers to significance with *p* < 0.05.

**Table 2:** The peak vertical ground reaction force (VGRF) and vertical instantaneous loading rate (VILR) during the landing phase between forward landing and backward landing.

	FS	BS	*p*	FD	BD	*p*
Peak Vertical Ground Reaction Force (BW)	4.11 ± 0.17 *	4.78 ± 0.16 *	0.001	2.61 ± 0.12	2.69 ± 0.18	0.533
Vertical Instantaneous Loading Rate (BW/S)	190.26 ± 11.58 *	217.97 ± 14.16 *	0.041	133.96 ± 4.51	125.76 ± 12.09	0.322

Note: BW: Body Weight, BW/S: Body Weight per second. * refers to significance with *p* < 0.05.
